# Career adapt-ability scale -short form: Validation among early career stage of Chinese university graduates

**DOI:** 10.3389/fpsyg.2023.1110287

**Published:** 2023-01-27

**Authors:** Chunze Song, Shahabuddin Bin Hashim, Xinpeng Xu, Hairong Ling

**Affiliations:** ^1^School of Educational Studies, Universiti Sains Malaysia, Pulau Pinang, Malaysia; ^2^General Education Center, Communication University of China, Nanjing, China; ^3^School of Public Health, Nanjing Medical University, Nanjing, China; ^4^Institute of Healthy Jiangsu Development, Nanjing Medical University, Nanjing, China; ^5^School of Education Science, Nanjing Normal University, Nanjing, China

**Keywords:** CAAS-SF, career adaptability, scale verification, early career stage, Chinese university graduates

## Abstract

The Career Adapt-Ability Scale (CAAS) is the favored method among researchers for measuring career adaptability. The 12-item version of CAAS-SF, which was made by Maggiori, Rossier, and Savickas based on a change to CAAS, has been slowly used by different groups in different countries and regions. As samples for the validation of the scale in this study, 571 Chinese university graduates in the early stages of their professions were chosen. Principal component analysis and confirmatory factor analysis suggest that CAAS-SF and CAAS have very similar psychological measurement features and factor structures. And the internal consistency of each subscale and total scale are equivalent to or greater than that of the CAAS assessment. These findings indicate that the CAAS-SF is a valid and reliable instrument for evaluating China’s career adaptability. In addition, limitations, issues for further research, and suggestions are emphasized.

## Introduction

Research on career adaptability is one of the most significant discoveries in occupational psychology during the past three decades (Nye et al., 2017), which has been further developed The notion of career adaptability has been embraced by scholars in the career area, as seen by the growing number of studies published on the topic over the past several years (Johnston, 2016).

At the same time, with the quick iteration and update of the technology, the fast and rapid growth of society, and the regularly changing conditions of life and work, individual flexibility and adaptation have become extremely important and are seen as a prerequisite for success (Sullivan and Baruch, 2009;Johnston, 2016). The idea of career adaptability is commonly seen as an essential resource for effective career development, good responses to career obstacles, and the increase of individual satisfaction (Zacher, 2014; Chan and Mai, 2015; Johnston et al., 2015; Johnston, 2016). Faced with the terrible worldwide impact of COVID-19, people’s everyday lives and careers have been significantly damaged, with ominous consequences for their futures (Akkermans et al., 2020). The existence of unemployment is pervasive, and the number of empty new roles is decreasing. The diminishing employment market has a significant effect on individual careers, particularly those of college students (ILO, 2020). Therefore, a rapid grasp of individual career adaptability in the early stages of one’s career should be of great assistance to the career counseling of university faculty to students and the career guidance of new workers in a variety of firm divisions. This is a win-win situation for early-career individuals, colleges, and associated businesses.

At present, there are now two comparable studies in China. One of the studies was conducted primarily among Macao employees who just graduated from the same public university (Sou et al., 2020), and hence their adaptability to the vocation is fairly limited. The second research focused on students from four universities and several limited industries including government, manufacturing, information technology, and service (Yu et al., 2019). Consequently, the representativeness of the study population is limited. The study aims to expand the diversity of the study groups in order to make results more representative for career development guidance. Considering that there are certain differences between China and other countries and regions in student origin region, college classification and specialty setting, it is hoped to explore the equivalence of these demographic factors in measuring career adaptability in the study.

## Career adaptability

Career adaptability is a concept conceptualized by Super, an American career planning master, revises his career maturity theory. Based on the classic understanding of individual variations in vocations, Super added the perspective of career development. In his career development theory proposed in 1955, Super first utilized the central notion of occupation maturity (Super, 1955). Then, in 1965, Crites established the concept of professional maturity, which supplanted this notion (Crites, 1974). Super and Knasel presented for the first time the notion of “career adaptability” after discovering that career maturity had significant limitations in understanding adult career growth (Super and Knasel, 1981). The concept of career maturity is gradually being replaced by the concept of career adaptability (Super et al., 1988). They believed that the concept of resilience highlighted the interaction between individuals and the environment and was more suitable for examining the career development of adults.

In the 1980s and 1990s, Pratzner (1978), Goodman (1994), Savickas (1997), and other researchers examined career adaptability and the significance of career adaptability in professional development concerns. On the basis of Super’s theoretical contributions, Savickas (1997) enhanced the theory of career adaptability from a career constructivist perspective. He thought that individuals were subjectively responsible for their own job progress. And they were able to actively investigate and build personal career tasks through career adaptation, which had four components: self-exploration, environmental exploration, career planning attitude, and career decision-making. Individual career adaptation is the capacity to successfully integrate individual career positions in order to attain career success. According to him, Super’s theory of career development would be more comprehensive if career adaptability were the central concept since career maturity emphasizes that individuals exist in a fixed environment, whereas career adaptability highlights the interaction with both individuals and their environment. Clearly, this concept is more in accordance with the requirements of the current era (Savickas, 1997).

Through a theory-evidence-theory method, Savickas has considered and studied career adaptability in a variety of ways and depth, and he has now created a more comprehensive theoretical framework: Career Construction Theory (CCT) (Savickas, 1997, 2002, 2005, 2013). Career adaptability is an essential component and fundamental notion of CCT theory (Johnston, 2016; Yu et al., 2019). Initially, according to the career maturity theory (Super and Knasel, 1981), Savickas investigated and presented a three-dimensional adaptive construct consisting of planful attitudes, self and environmental inquiry, and adaptive decision-making (Savickas, 1997). On the basis of the Social Cognitive Career Theory, Savickas subsequently added the dimension of Career confidence and changed it into a four-dimensional theoretical structure (Savickas, 2002; Savickas and Porfeli, 2011). In 2005, Savickas refined and enhanced the CCT theory of career adaptability, claiming that career adaptability develops along four dimensions, including career concern, career control, career curiosity, and career confidence. Among these, career concern refers to an individual’s interest in his or her future career development. Career control refers to the subjective initiative and liberty of the individual in pursuing future career development. Career curiosity refers to the active pursuit and modification of career self-cultivation and employment options during an individual’s career development, as opposed to passive development. Career confidence in one’s career refers to the fortitude and trust an individual has in overcoming obstacles and achieving career goals. Therefore, according to Savickas, career adaptability is a type of psychological resource that enables individuals to deal with and mobilize psychological energy when confronted with career responsibilities, issues, and changes, along with career crisis events during the career growth process (Savickas, 2005; Savickas and Porfeli, 2012).

Acceptance and expansion of the notion of career adaptability amongst researchers is a lengthy process. In the present research, career adaptability has received increasing attention, particularly in the widely utilized study on self-career management and individual career development.

## Instruments for assessing career adaptability

Johnston (2016) uncovered eight primary instruments for evaluating career adaptability through a thorough assessment of the literature. CAAS (Savickas and Porfeli, 2012), CAAS-SF (Maggiori et al., 2015), CWAQ (Nota et al., 2012), CMI-Form C (Savickas and Porfeli, 2011), CAI (Ferreira, 2013; Ferreira et al., 2013), SCCI (Savickas, 2009), CFI (Rottinghaus et al., 2005) and the I-Adapt Scale (Ployhart and Bliese, 2006). CWAQ, CMI-Form C, CAI, and SCCI are mostly focused upon Savickas’ concept of career adaptability and attempt to quantify comparable or very similar characteristics. CFI and IAS are measured from different perspectives, but their applicability is limited (Johnston, 2016). The CAAS (Savickas and Porfeli, 2012) is the most widely used scale (Yu et al., 2019; Pal and Jena, 2021). The Career Adapt-Ability Scale (CAAS) is a cross-cultural international version created by Savickas and a worldwide research group comprised of professionals and scholars from 18 nations and regions. Every single of the dimensions of concern, control, curiosity, and confidence was measured with six questions, for a total of twenty-four questions (McMahon et al., 2012; Savickas and Porfeli, 2012). The CAAS scale has been tested in 13 countries and regions from Eastern and Western, as well as having survived the consistency test in a wide range of cultural situations, demonstrating its broad application (Chen et al., 2020).

Due to the pressures of school, life, and job, the shorter the questionnaire, the less burdensome it is to respond, and the greater the degree of completion (Işık et al., 2018). In particular, using a shorter scale allows researchers to evaluate the relationship between career flexibility and other research parameters concurrently (Maggiori et al., 2015; Pal and Jena, 2021). Therefore, a relatively compact and effective measurement device is necessary (Johnston, 2016; Yu et al., 2019). Additionally, a short scale is especially useful in large panel study or in survey investigating numerous aspects.

In this regard, Maggiori et al. (2015) produced a 12-item form of the CAAS-SF. In the last four years, validation studies on the application of CAAS-SF in some nations have determined that the 12-item CAAS-SF (Maggiori et al., 2015) has a strong correlation with the 24-item CAAS (Savickas and Porfeli, 2012). In Australia (Perera and McIlveen, 2017), Turkey (Işık et al., 2018), Switzerland (Urbanaviciute et al., 2019), China (Yu et al., 2019), Germany (Haenggli and Hirschi, 2020), and India (Pal and Jena, 2021), similar results were found in related studies, and both the reliability as well as the validity of the measure were good. In these studies, it was observed that although the scale was halved in size, the 12-item CAAS-SF displayed the same component structure and psychometric qualities as the 24-item variant. (Maggiori et al., 2015; Işık et al., 2018; Yu et al., 2019; Sou et al., 2020; Pal and Jena, 2021). The aim of this study was to validate the Chinese version of the 12-item CAAS in a large sample among early career stage of Chinese university graduates.

## Materials and methods

### Participants

This study focuses primarily on graduates of colleges and universities in Jiangsu Province or those who work in Jiangsu Province, with no restrictions on the types of institutions, majors, or units. Participants in the questionnaire survey are recent graduates who have worked for three months to five years.

62.52 percent of the 571 valid data obtained were female, and 46.58 percent were from rural areas. 101 people (17.69%) were college graduates, 384 people (67.25%) had a bachelor’s degree, and 86 people (15.06%) had a master’s or doctoral degree. 474 (83.02%) of them graduated from undergraduate schools, and 97 (16.98%) from colleges; 197 (34.50%) majored in literature, 204 (35.73%) in science and engineering, and 170 (29.77%) in art and sports. From 2017 through 2021, the proportions of graduates were as follows: 21.19 percent, 11.38 percent, 12.96 percent, 23.12 percent, and 31.35 percent. The respondents work in 26 provinces in China, with Jiangsu (54.47%), Shanghai (12.26%), and Beijing (4.72%) ranking as the top three. In the meanwhile, there are a few individuals who work in nations other than China. The participants came from a variety of occupational backgrounds, including but not limited to teachers, doctors, administrators, technology developers, freelancers, and more.

### Procedure

The collection of samples in this study was approved by the Scientific Research Office of Communication University of China, Nanjing. This study was done using an online poll with non-random sample methods, including convenience sampling and snowball sampling. In this study, Internet invitations to complete questionnaires were sent out. The graduates, coworkers, and alumni who satisfied the survey’s qualifications were invited to fill out the survey online; they were also encouraged to invite acquaintances or colleagues who also meet the survey’s conditions to participate. The participation was entirely voluntary. The goal of the research is explained to each willing participant, and confidentiality and anonymity of the data are assured.

In November 2021, the test link was distributed to the appropriate staff and 700 surveys were sent out and gathered. The questionnaire would be regarded as invalid, if the answer time is too short (less than 5 min). After screening and removing invalid questionnaires, 571 valid questionnaires were recovered, representing an effective recovery rate of 81.57%.

### Measures

The 12-item CAAS-SF scale updated by Maggiori et al. (2015), which was derived from the 24-item CAAS (Savickas and Porfeli, 2012) produced by Savickas et al., was used to evaluate career adaptability in this study. The English wording of the measurement issue has been modified in accordance with the findings of this study and local reading preferences. In addition, this study cites the Chinese version of the Career Adapt-Abilities Scale-China Form amended by Hou (Hou et al., 2012).

The scale retains four aspects: career concern, career control, career curiosity, and career confidence. Each test aspect consists of three exam items, for a total of twelve. Adoption of the Likert5-level scoring approach. “1 = extremely inconsistent, 2 = moderately inconsistent, 3 = unsure, 4 = moderately consistent, 5 = extremely consistent.” The particular elements are detailed in Table A1.

### Analyses plan

IBM SPSS Statistics 26 was used for data entry and analysis in this study. On the research data, descriptive statistics, the non-parametric hypothesis test, the Spearman correlation analysis, the reliability test, as well as the exploratory factor analysis were performed. Using the statistical software IBM SPSS AMOS 26 for structural equation modeling analysis, being can conduct confirmatory factor analysis.

In each analysis, different indicators have different cutoff values. In the majority of the reliability test, Cronbach’s α coefficient and the CITC value of each item should be more than 0.70 and 0.50, respectively (Eisinga et al., 2012). In the validity analysis, the KMO value must be greater than 0.80 and the estimated value of the Bartlett sphere must be greater than 0, followed by factor analysis if both conditions are satisfied. In factor analysis, structural validity evaluates whether the load of each item on the related factor exceeds 0.40 and the degree of the common factor exceeds 0.50 (Pallant, 2010). The convergent validity requires that the AVE value of each factor exceeds 0.50 and the CR value exceeds 0.70. If the AVE value of each component is bigger than the “highest correlation coefficient between the factor and other factors,” then discriminative validity is present (Gim Chung et al., 2004). In terms of model fitting index, when χ^2^/df is equal to or less than 5 (Bollen, 1989), CFI, TLI, IFI, and NFI are typically acceptable between 0.90 and 0.95, and values greater than 0.95 suggest successful model fitting (Hu and Bentler, 1999; Maggiori et al., 2015). RMSEA less than 0.05 indicates an excellent fit, whereas RMSEA between 0.05 and 0.08 indicates an appropriate fit (Browne and Cudeck, 1992).

## Result

### Descriptive statistics and reliability estimates

Reliability Analysis Results of CAAS-SF China and the Relevant Data of the Observation Variable (Table 1) demonstrates that the overall structure of CAAS-SF has a dependability of 0.944, which is more than that of concern (0.869), control (0.849), curiosity (0.867), and confidence (0.887). The Cronbach alpha coefficients of each factor dimension and the total exceed the empirically acceptable cutoff value of 0.70 (Nunnally, 1978), which indicates that the reliability quality of the research data is very high. For the “Cronbach’s α of deleted item,” after any item is deleted, the reliability coefficient will not increase obviously, indicating that all items should be kept. According to the “CITC,” the CITC value of each analysis item is greater than 0.50, which indicates that all analysis items have a good correlation, and the reliability level is good (Eisinga et al.,2013).

**Table 1:** Reliability Analysis Results of CAAS-SF China and the Relevant Data of the Observation Variable.

Variable	Potential variable	Observation variable	Mean	SD	Std. Estimate	Coef.	CITC	Cronbach’s α of deleted item	Cronbach’s α	
Adaptability	Concern	CA1. I think about what my future will be like.	3.91	0.86	0.82	1.05	0.737	0.940	0.869	0.944
		CA2. I Prepare for the future.	3.83	0.90	0.86	1.16	0.728	0.940		
		CA3. I’m aware of the educational and vocational choices that I must make.	3.82	0.82	0.82	1.00	0.700	0.941		
	Control	CA4. I make decisions by myself.	3.92	0.85	0.76	0.98	0.674	0.942	0.849	
		CA5. I take responsibility for my actions	4.14	0.80	0.86	1.03	0.769	0.939		
		CA6. I count on myself.	4.04	0.83	0.80	1.00	0.723	0.940		
	Curiosity	CA7. I look for opportunities to grow.	4.01	0.77	0.86	1.03	0.798	0.938	0.867	
		CA8. I investigate options before making a choice.	4.04	0.80	0.84	1.04	0.760	0.939		
		CA9. I observe different ways of doing things from people around.	4.02	0.83	0.78	1.00	0.721	0.940		
	Confidence	CA10. I take care to do things well.	4.14	0.78	0.82	0.97	0.764	0.939	0.887	
		CA11. I learn new skills.	4.05	0.81	0.89	1.09	0.785	0.938		
		CA12. I develop my ability step by step.	4.07	0.76	0.86	1.00	0.769	0.939		

Data source: Collated through this study.

### Factor analysis

Prior to the CAAS-SF factor analysis, the scale’s appropriateness for factor analysis was evaluated. The value of KMO is 0.94, the Bartlett chi-square number is 5081.765, and the *p*-value is around 0, *p* < 0.001. So, CAAS-SF is eligible for factor analysis. Through principal component analysis, four factors were recovered for exploratory factor analysis. Table 2. The EFA Results of CAAS-SF China display the factor load matrix for each measurement item. The KMO value of this scale was 0.944 > 0.6, the common degree value of all items was greater than 0.5, the explanation rate of variance for each factor after rotation was 21.75, 18.90, 18.84, and 20.61%, respectively, and the explanation rate of cumulative variance was 80.10% > 50%. All of these indicate that the information about the study item can be successfully articulated, and that the measured data is extremely suitable for information extraction and can be extracted effectively, indicating good validity on the side (Gim Chung et al., 2004).

**Table 2:** The EFA Results of CAAS-SF China.

Observation variable	Factor load coefficient				Common degree (common factor variance)
	Concern	Control	Curiosity	Confidence	
CA2	0.813	0.252	0.231	0.230	0.832
CA3	0.810	0.233	0.193	0.235	0.802
CA1	0.710	0.288	0.278	0.272	0.738
CA4	0.333	0.814	0.223	0.104	0.835
CA6	0.240	0.710	0.207	0.399	0.764
CA5	0.245	0.672	0.350	0.374	0.774
CA8	0.296	0.275	0.780	0.271	0.846
CA9	0.189	0.281	0.740	0.345	0.781
CA7	0.439	0.222	0.625	0.387	0.782
CA11	0.325	0.250	0.277	0.787	0.865
CA12	0.279	0.189	0.407	0.741	0.827
CA10	0.249	0.381	0.297	0.685	0.765
Sampling suitability measure		KMO	0.944		
Bartlett test		c^2^	5081.765		
		df	66		
		p	0		
Variance explanation rate (after rotation)	21.75%	18.90%	18.84%	20.61%	-
Cumulative variance explanation rate (after rotation)	21.75%	40.65%	59.49%	80.10%	-

Data source: Collated through this study.

**Figure 1:**
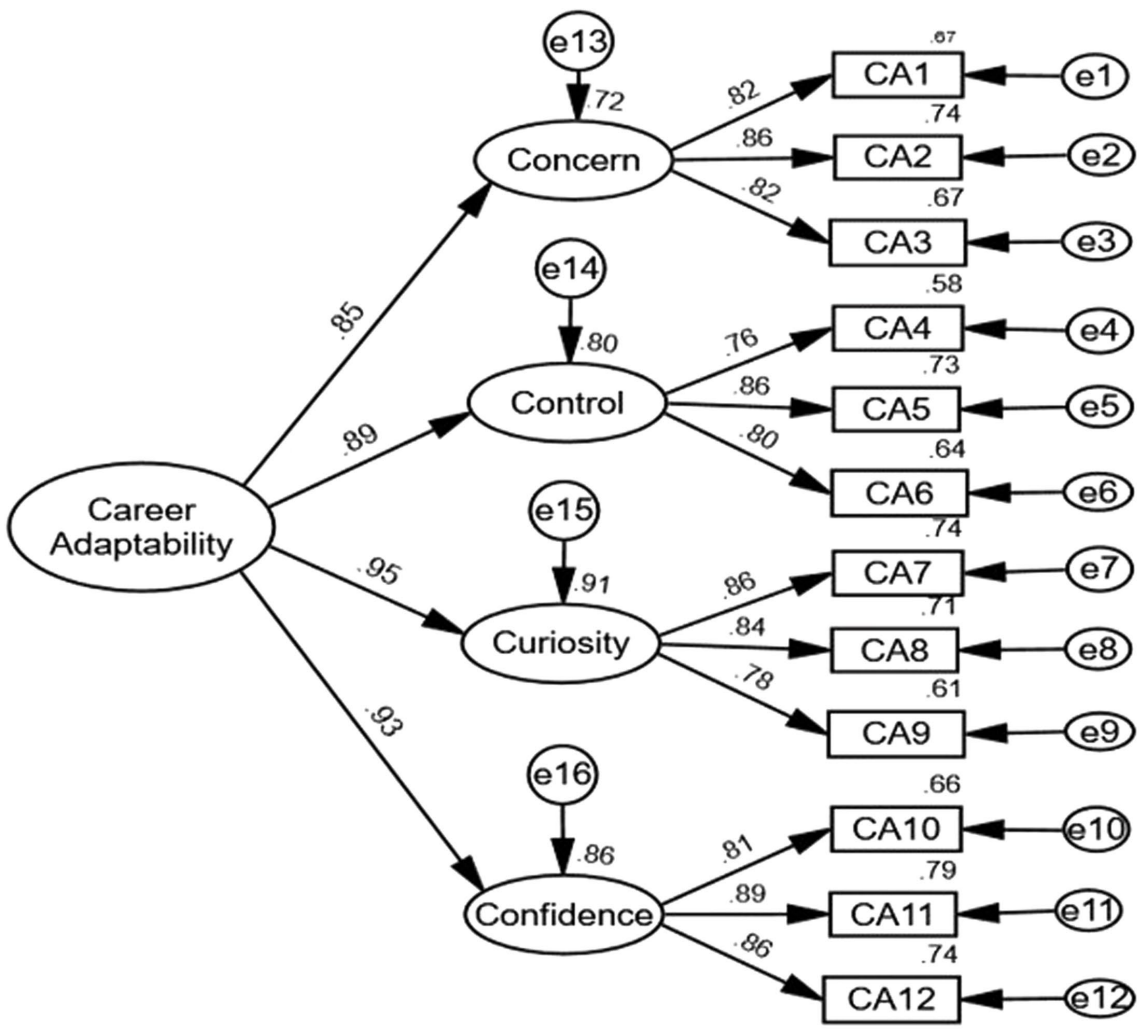
Confirmatory Factor Analysis of Career Adapt-Abilities Scale–Short Form China. Note. N = 571. Standardized regression coefficients are shown. Source: Collated through this study.

The CFA was performed on a total of four components and twelve analysis questions. Combined with Table 3. The CFA Results of CAAS-SF China, AVE square root values of the 4 factors of CAAS-SF were 0.831, 0.808, 0.829, and 0.854, respectively, which were greater than the maximum values of absolute correlation coefficients of 0.697, 0.723, 0.793 and 0.793 of the corresponding factors. It means that the four factors have good discriminative validity (Gim Chung et al., 2004). AVE values referring to each factor are all larger than 0.5 (Fornell and Larcker, 1981), and combined reliability CR values all seem to be greater than 0.7 (Hair et al., 2019), indicating that the convergent validity of the analytical data gathered by this scale is good. From the perspective of measurement relationship (Referring to Figure 1. Confirmatory Factor Analysis of CAAS-SF China), the absolute value of the standardized load system of each measurement relationship is greater than 0.7 and presents a significant significance, indicating that there is a good measurement relationship (Gim Chung et al., 2004).

**Table 3:** The CFA Results of CAAS-SF China.

Factor	AVE and CR index results of the model		Pearson correlation and AVE square root value			
	AVE	CR	Concern	Control	Curiosity	Confidence
Concern	0.691	0.871	0.831			
Control	0.653	0.849	0.682	0.808		
Curiosity	0.687	0.868	0.697	0.713	0.829	
Confidence	0.730	0.890	0.681	0.723	0.793	0.854

Note: The diagonal blue numbers are the square root values of AVE. Data source: Collated through this study.

As shown in Figure 1. Confirmatory Factor Analysis of CAAS-SF China, in this Chinese sample, the factor loadings of the 12 first-order items were comparable to the data presented in the original CAAS-SF (Maggiori et al., 2015), with ten items showing higher factor loadings than the original CAAS-SF, while item 4 (CA4, 0.76) and item 12 (CA12, 0.88) had lower factor loadings than the original CAAS-SF. Compared to the three data from the same Chinese sample in another study (Yu et al., 2019), all 12 items in the civil servant sample had larger loadings than the samples of students and ordinary employees, with the exception of one item. Concern (0.85), control (0.89), curiosity (0.95), and confidence (0.93), which were greater than the original CAAS-SF values of 0.73, 0.75, 0.88, and 0.77, but in the same order, were the loading values of the second-order adaptive structure in the data of this study. The concern factor loading is less than that of India (0.94). However, when compared to other Chinese academicians (e.g., Sou et al., 2020), the values were more similar.

### Structural model fitting analysis

According to the fitting index results of structural model, the fitting value of the structural model is χ^2^/df = 3.739, slightly over the cut-off value 3 and lower than 5 (Bollen, 1989), and RMSEA = 0.069, which is between 0.05 and 0.08, while CFI (0.973), TLI (0.964), IFI (0.973), and NFI (0.974) are all higher than 0.9 (Browne and Cudeck, 1992; Hu and Bentler, 1999). In general, the model fitting effect is satisfactory, indicating that it has strong applicability, and it supports the use of this scale in the early career stage of Chinese college and university graduates.

### Demographic correlation analysis

Using chi-square analysis for possible differences between demographic profiles, CAAS-SF scores varied with gender (χ^2^ = 47.589, *p* = 0.114), place of origin (χ^2^ = 39.179, *p* = 0.372), educational background (χ^2^ = 70.810, *p* = 0.584), school category (χ^2^ = 110.848, *p* = 0.496), major category (χ^2^ = 82.363, *p* = 0.237) and graduation time (χ^2^ = 154.428, *p* = 0.342) had no statistical significance.

## Discussion

On the basis of the statistical analysis of the sample of Chinese university graduates in the early stages of their careers, it is possible to conclude that the findings of this study concur with those presented by Maggiori et al. (2015). Comparing the psychometric properties and factor structure of CAAS-SF to other worldwide samples from recent years reveals a high degree of similarity. In terms of data specifics, the Cronbach alpha coefficient indicated that each subscale’s coefficient value was greater than 0.8, and the total coefficient reached 0.94. Comparable to samples from other countries, the internal consistency of the total programme and the four sub-dimensions was strong in China’s usage of the CAAS-SF. In the meantime, the results of EFA’s main component analysis and CFA’s confirmatory analysis both corroborate the four-factor dimension structure of CAAS-SF and are compatible with the dimension structure of CAAS. Therefore, this study confirms the applicability and validity of the questionnaire among Chinese university graduates at their early career.

From the results of the confirmatory factor analysis of this sample data, the curiosity’s factor loading rated first (0.95), and the confidence’s factor loading ranked second (0.93), both of which were greater than those of concern (0.85) and control (0.89). The values of the four components are greater than those measured by the original CAAS-SF (2015), but their overall trend is consistent. This is also comparable with Sou’s research on recent graduates in Macao, China (Sou et al., 2020), with a higher degree of consistency to Sou’s research on size and trend. In contrast, Pal’s study in India revealed that curiosity was the least important of the four criteria. He speculated that this disparity may reflect divergent perspectives on competition, unemployment, and the intense pressure to find suitable employment (Pal and Jena, 2021). This may be mostly attributable to regional, cultural, economic, and other variables. This must be confirmed by other researches. These distinctions will also be the subject of future investigation.

There are further intriguing findings from the study. In terms of gender, for instance, the findings of this study differ from those of prior research conducted in China (e.g. Yu, 2008; Zhao and Guo, 2010; Hou et al., 2012; Yu et al., 2019). Throughout this study, the gender among Chinese university graduates at the beginning of their careers had no effect on their adaptability. However, according to the findings of Hou et al. (2012), Yu et al. (2019), and other researchers, males in China are perceived to be more versatile in their jobs than women. They believe this is related to conventional gender norms and typical gender labor distribution within China, where men were commonly viewed as bound to the workplace and women to domestic duties (Hou et al., 2012; Yu et al., 2019). This may be because, as a result of societal progress, women’s education and ability training have vastly improved, and their participation in the workplace and the level of attention have increased. In addition, there is no distinction between school type and adaptability to the workplace. Under the policy and direction of the national education department, schools at all levels place a greater emphasis on teaching and training in pertinent areas to increase students’ vocational adaptability. This is in relation to the cultivation of vocational adaptability.

### Theoretical and practical implications

This study builds upon the work of previous scholars. This article focuses primarily on expanding the research object in China, covering the population structure and occupation structure. This study’s selection of research objects differs in several ways. Previous studies primarily focused on university students or university students about to graduate or a specific working group (Yu et al., 2019; Sou et al., 2020; Pal and Jena, 2021). However, the former are still students and not actual workers, and this may impact their perception of the state of their careers. The latter is exclusive to a certain working group, and the sample size is insufficient. This study’s selection of research objects will expand the employment field and scope of Chinese university graduates, increase the representation of the research group, provide a new supplement to the verification field of CAAS-SF in China, and bolster the applicability of verification of vocational construction theory in various social groups.

### Limitations and future implications

This work has certain remaining shortcomings, which indicate to the path of future research. First, although the professional area and age range of research subjects have been broadened, this is still a cross-sectional study; therefore, future research should focus on the longitudinal link between research variables. Second, the current sample comprises graduates of different majors from four different types of institutions in China, but Jiangsu is the main province, which must be expanded to other provinces to improve the quality and representativeness of the research data. Thirdly, the reliability and validity of the scale can be confirmed further by taking into account various cultural backgrounds, regional characteristics, and economic conditions. Furthermore, career adaptability is the application of adaptability in the field of work and is not a comprehensive evaluation of an individual’s adaptability; those experiencing job transitions may be more influenced by career adaptability. The selection of the study population and sampling techniques for career adaptability research could be further refined in future studies.

## Conclusion

In conclusion, CAAS-SF is appropriate in the Chinese setting. This research also provides assistance with the implementation of CAAS-SF in China’s early career stages and across a variety of occupational fields. Through the implementation of CAAS-SF, it will be easier and faster to comprehend the relationship between the professional adaptability of Chinese college graduates in their early career stage and other pertinent study variables.

## Data availability statement

The raw data supporting the conclusions of this article will be made available by the authors, without undue reservation.

## Ethics statement

The studies involving human participants were reviewed and approved by The Scientific Research Office of Communication University of China, Nanjing. Written informed consent for participation was not required for this study in accordance with the national legislation and the institutional requirements.

## Author contributions

SH and XX supervised the topic selection and research design. CS put forward the core point of this research and wrote the paper. HL was responsible for data collection and modifying the manuscript. All authors contributed to the article and approved the submitted version.

## Funding

This study was funded by the Philosophy and Social Science Research Project of Universities in Jiangsu Province, Project no. 2020SJB1184.

## Conflict of interest

The authors declare that the research was conducted in the absence of any commercial or financial relationships that could be construed as a potential conflict of interest.

## Publisher’s note

All claims expressed in this article are solely those of the authors and do not necessarily represent those of their affiliated organizations, or those of the publisher, the editors and the reviewers. Any product that may be evaluated in this article, or claim that may be made by its manufacturer, is not guaranteed or endorsed by the publisher.
